# Differences in the Incidence of Symptomatic Cervical and Lumbar Disc Herniation According to Age, Sex and National Health Insurance Eligibility: A Pilot Study on the Disease’s Association with Work

**DOI:** 10.3390/ijerph15102094

**Published:** 2018-09-25

**Authors:** Young-Ki Kim, Dongmug Kang, Ilho Lee, Se-Yeong Kim

**Affiliations:** 1 Department of Occupational and Environmental Medicine, Pusan National University, Yangsan Hopspital, Yangsan, Mulgeum-eup, Bumeo-ri, Yangsan, Gyongnam 626-770, Korea; mungis@pusan.ac.kr (Y.-K.K.); kangdm@pusan.ac.kr (D.K.); ihlee84@naver.com (I.L.); 2 Department of Preventive and Occupational & Environmental Medicine, School of Medicine, Pusan National University, Busan, Mulgeum-eup, Bumeo-ri, Yangsan, Gyongnam 626-770, Korea

**Keywords:** disc herniation, age, sex, insurance eligibility

## Abstract

The aim of this research was to identify the differences in the incidence of symptomatic cervical and lumbar disc herniation according to age, sex, and national health insurance eligibility. We evaluated the hospital documents of patients who received medical treatment for symptomatic cervical and lumbar disc herniation between 2004 and 2010 and excluded those who claimed to have expenses at oriental medical clinics or pharmacies. Furthermore, any duplicate documents from the labor force population aged 20–69 years were excluded from the analysis. The results showed that the number of individuals diagnosed with symptomatic cervical and lumbar disc herniation increased with age, and the incidence of these diseases was higher in women than in men. Additionally, the incidence differed depending on the subject’s qualification for health insurance. The incidence of lumbar disc herniation showed differences depending on the degree of the lumbar burden. The present study findings may help determine whether lumbar disc herniation is associated with tasks performed at the patient’s workplace. Further research is needed to classify the risk of lumbar disk herniation in the workplace into detailed categories such as types of business, types of occupation, and lumbar compression force.

## 1. Introduction

Disc herniation (DH) is the displacement of disc material (nucleus pulposus or annulus fibrosis) beyond the intervertebral disc space [1]. Lumbar disc herniation (LDH) is the major cause of morbidity and its treatment is very expensive [2]. In South Korea, DH greatly affects a worker’s compensation. It has also been considered a controversial disease for medical compensation approval because it is difficult to medically identify whether DH is caused by occupational factors or factors naturally developed by individuals [3]. Although there is no basic epidemiological information for determining whether DH is associated with occupational factors, some epidemiological studies have revealed the incidence of DH. According to Jordon, the prevalence of LDH in Finland and Italy is 1–3%, which is higher for individuals aged 30–59 years, with men having a two times higher incidence than women [1]. Deyo et al. assumed that the incidence of LDH in America would reach about 1–2% [2]. Radhakrishnan et al. reported that the annual incidence of cervical disc herniation (CDH) in Rochester and Minnesota between 1976 and 1990 reached its peak for individuals in their 60s, with 18.6 of 100,000 individuals developing the disease [4]. Meanwhile, a significant number of asymptomatic disc herniation (DH) cases may be present as well [5,6]. However, these results have been limited to a certain age group, region, occupation, or group and cannot be referred to when evaluating the occurrence distribution in all age groups or the differences in DH incidence between men and women according to age [7,8,9,10,11,12,13,14], thereby limiting their generalizability. The limitations of these epidemiological studies are attributed to the lack of analysis of nationwide diagnosis documents which until now had been impossible. However, since big data analysis has become a major aspect of medical studies, several attempts have been made to analyze all health insurance data and identify the distribution of disease occurrence [15]. In South Korea, health insurance data have become public, enabling the study of the distribution of disease occurrence through analysis of the data from the whole country.

Generally, identifying the distribution of disease occurrence is crucial for preventing a disease and determining the association between occupational factors and disease occurrence in order to determine worker’s compensation. Therefore, in this study, we analyzed health insurance data to identify the distribution of symptomatic CDH and LDH occurrence according to age, sex, and workforce eligibility for national health insurance. Asymptomatic DH was excluded from the analysis as its presence could not be determined from health insurance data. The qualification for health insurance varies with the characteristics of the tasks performed in the workplace. The reason for investigating the distribution of incidence by eligibility was to determine whether there were differences in the incidence of DH according to type of labor, as the nature and type of labor performed by the subscribers may vary according to their eligibility.

## 2. Materials and Methods

### 2.1. Study Subjects

We obtained health insurance big data from the National Health Insurance Service in Korea. Then, data on subjects who were diagnosed with CDH and LDH and received medical treatment between 2004 and 2010 were selected at the headquarters of a big data analyzing laboratory. To select the subjects, the following diagnosis codes were used: M500 (cervical disc disorder with myelopathy), M501 (lumbar and other disc disorders with myelopathy), M502 (other cervical disc displacement disorders), M510 (lumbar disc disorder with myelopathy), M511 (herniated disc disease of the lumbar spine with radiculopathy), and M512 (herniated intervertebral disc).

### 2.2. Methods

Among the study subjects who were selected by disease codes, subjects for whom the accuracy of diagnosis could not be confirmed, such as those who had been to a pharmacy or an oriental medical clinic, were excluded. However, the health insurance data represent claims data for insurance payments, and if a person received treatment for DH on multiple occasions, then the number of times that person was diagnosed with DH would be calculated in duplicate. Accordingly, to prevent duplicate calculation for a single subject, all records, except the initial hospital diagnosis of CDH or LDH were deleted. In addition, to identify the relevance of occupational factors, subjects were limited to those aged 20–69 years because they represent the actual labor population; subjects in other age groups were excluded. Those who were eligible for health insurance consisted of regional subscribers, company subscribers, government office and private school subscribers, and medical care subscribers. Regional subscribers included those who own their own businesses, such as a restaurant or beauty salon, and perform various forms of physical labor. Company subscribers included those who were employed in companies; approximately 70% of company subscribers performed field work that required physical work, whereas the major duties of the remaining 30% involved office work. Most of the government office and private school subscribers performed office work. Most of the medical care subscribers performed physical work with low payment, such as daily construction site work. In addition, health insurance data did not include worker’s and military compensation.

As the study population size varied by subscriber type, sex, and age, the number of individuals diagnosed with CDH and LDH was calculated by using the total number of individuals diagnosed with CDH and LDH between 2004 and 2010 as the numerator and the total number of individuals over same 7-year period by age, sex, and health insurance eligibility as the denominator. The results were represented as the number of individuals diagnosed with these diseases out of 100,000 individuals in a period of 7 years.

### 2.3. Statistical Analysis

For the statistical analysis, SAS version 9.2 (SAS Institute, Cary, NC, USA) was used along with data cleaning. Frequency analysis and the chi-square test were also performed.

### 2.4. Ethics Statement

The present study protocol was reviewed and approved by the Institutional Review Board of Pusan National University, Yangsan Hospital College (approval No. 04-2015-008).

## 3. Results

### 3.1. Mean Number of Subscribers by Gender, Age, and National Health Insurance Eligibilty

To determine the size by gender, age, and eligibility, the mean number of subscribers over the seven-year period from 2004–2010 is presented in Table 1. There were statistical differences between distribution by sex and subscriber type (*p* < 0.01).

### 3.2. Number of Examinees with CDH According to Gender, Age and Health Insurance Eligibility 

**Figure 1:**
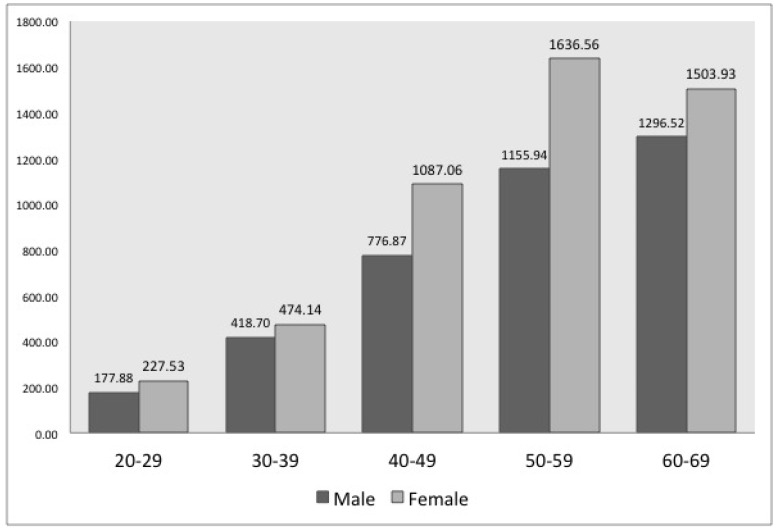
Number of patients with cervical disc herniation (CDH) of 100,000 individuals according to gender and age (2004–2010) *. * According to the Chi-square test, there was a statistically significant difference of frequency with respect to gender and age (*p*-value < 0.001).

The number of patients with CDH increased with age for both men and women. For every age group, the number of female patients was higher than that of male patients (see Figure 1). In terms of health insurance eligibility, the number of patients with CDH significantly differed between subscriber types (*p* < 0.01): The number of male patients with CDH who were regionally health insured was the lowest, while the rest of the male patients showed similar results. Meanwhile, the number of female patients with CDH was the highest for those subject to medical care, while the rest showed similar results (see Table 2).

### 3.3. Number of Patients with LDH According to Gender, Age, and Health Insurance Eligibility

**Figure 2:**
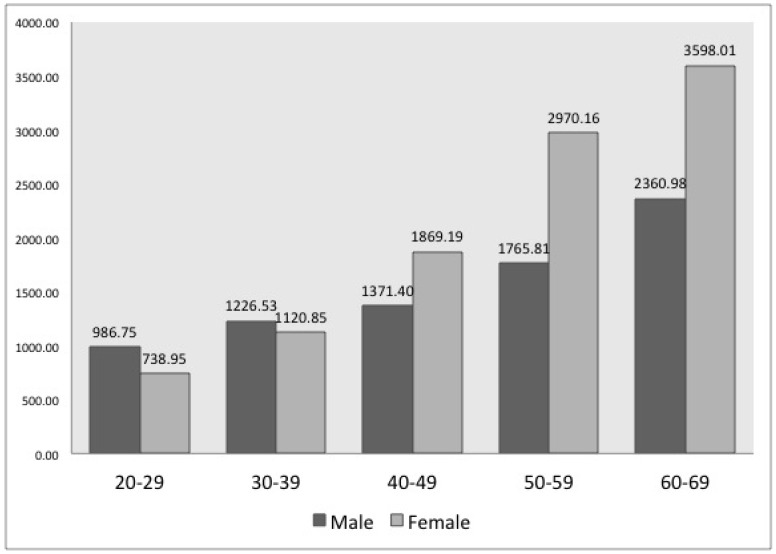
Number of patients with lumbar disc herniation (LDH) of 100,000 individuals according to gender and age (2004–2010) *. * According to the Chi-square test, there was a statistically significant difference of frequency with respect to gender and age (*p*-value < 0.001).

The number of patients with LDH increased with age for both men and women, similar to the results for those with CDH. However, the number of male patients with LDH was higher for those in their 30s, while there were more female patients with LDH aged ≥40 years (see Figure 2). In terms of health insurance eligibility, the number of patients with LDH significantly differed between subscriber types (*p* < 0.01); the number of male patients with LDH was highest for medical care subscribers, followed by company, government office and private school subscribers. Those who were regional subscribers had the lowest number of male patients with LDH. The number of female patients with LDH was the highest for medical care subscribers, followed by those who were covered by their company and regional subscribers, which had similar results. Those who worked at a government office or private school had the lowest number of female patients with LDH (see Table 3).

## 4. Discussion

Previous epidemiological studies on CDH and LDH were limited to a certain group of age, gender, occupation, or region; therefore, it is not possible to make a sound comparison between those studies and the present study, which analyzed data on DH from a whole country. However, to help readers understand this research, this study will be compared to previous ones.

The number of patients with CDH and LDH increased with age for both men and women. Generally, it is known that DH occurrence increases with age [16,17,18]. However, Jordon reported that the number of examinees was the highest for those aged 30–59 years [1], while Ma et al. revealed that the number of patients decreased for those aged ≥60 years [19]. These results are different from those of our study. In our study, as shown in Table 1, the number of subscribers decreased with an increase in age. However, the number of patients (male and female) diagnosed with DH in a population of 100,000 individuals increased with an increase in age (see Figure 1 and Figure 2). Moreover, although not reported in these study results, this increase was more distinct among patients aged ≥70 years than among those aged 60–69 years. 

In contrast to previous studies that demonstrated the number of patients was two times higher for men than for women [1,20], our study showed that the number of patients with CDH and LDH was higher in women than in men. Every age group of female patients with CDH and female patients with LDH above 40 years of age had a greater number than male patients, which can be interpreted as female patients being more genetically vulnerable to DH than male patients [21]. Our comparison between females and males by subscriber and work type revealed specific trends. For example, both female and male government office and private school subscribers in Table 2 and Table 3 are all office workers, indicating no difference in their work type, but the number of female patients was greater than the number of male patients in most age groups, except for patients with LDH in their 20s to 30s. The other subscriber types showed similar results. 

However, in the case of LDH, the number of male patients was higher than that of female patients below 40 years of age, which might be a consequence of the difference in tasks performed by men and women and the difference between the occurrence of CDH and LDH, rather than a naturally developed disease. As shown in Table 3, the number of LDH patients was lower among male medical care subscribers of all age groups and male regional subscribers of all ages except 20–29 years, than among female individuals, whereas number of LDH patients was appreciably higher among male company subscribers, male government office and private school subscribers in the age group 20–39 years than among female patients. The exact cause of this cannot be determined by the findings of our study. However, the higher levels of LDH diagnosis for men compared to women in these two subscriber types, when contrasted with other subscriber types, may be due to the type of labor performed by young male company subscribers, male government office and private school subscribers being affected by factors that can overcome genetic factors.

The occurrence of DH according to the category of health insurance eligibility showed different results with respect to the DH region and gender. Among males, the incidence of CDH, from high to low incidence, was as follows: company, government office and private school, medical care, and regional subscribers. Among females, the incidence of CDH, from high to low, was as follows: medical care, government office and private school, company, and regional subscribers.

Among males, the incidence of LDH, from high to low incidence, was as follows: medical care, company, government office and private school, and regional subscribers. In females, the number of patients with LDH, from high to low incidence, was as follows: medical care, company, region, and government office and private school subscribers. These distinctive results might be due to differences in the tasks performed or the relevance of the tasks. This is because if DH occurs due to genetic factors, it should not be possible to encounter differences according to health insurance eligibility, suggesting that acquired or environmental factors are responsible for these variations. Moreover, the physical factors account mostly for acquired or environmental factors [22]. Therefore, if occupational or non-occupational factors are to be compared, occupational factors account mostly for the consistency of work intensity and repeatability. For example, it is difficult to repeat a leisure activity, that is, a non-occupational factor for more than an hour a day, but people continually carry out work tasks for many hours a day over years. Therefore, it is clear that occupational factors account mostly for physical causes, which are more noticeable for LDH than for CDH. With respect to the medical care subscribers who belonged to the low-income group, which showed the highest amount of LDH, most people in this group often performed manual labor, such as daily construction site work. As a result, they were more likely to sustain lower back injuries. The next highest numbers were in those who worked at a company, of which 1/3 is comprised of office workers and 2/3 is comprised of manufacturing workers, indicating that manufacturing workers are at a higher risk of damaging their lumbar disc. These findings are consistent with the results of existing studies. In other words, previous study results have demonstrated that the onset rate of LDH increases with increasing physical labor and physical loads [23,24].

Overall, LDH showed a higher incidence rate than CDH. This seems to be attributable to the fact that the lumbar spine bears a greater load, as it bears the weight of both the head and upper extremities, whereas the cervical spine needs to support only the head. For CDH, posture is the main risk factor and there are very few differences between daily life and workplace postures. On the other hand, handling heavy loads, postural factors, and whole-body vibration are the most important risk factors for LDH. Accordingly, it has been hypothesized that the onset rate of LDH is higher than that of CDH because more types of loads have a greater impact on LDH than on CDH.

There are several limitations to this study. The data used in this study contain the medical expenses that the subjects reported to the Health Insurance Service. Therefore, it might contain inaccurate diagnosis data, in order to falsely claim medical expenses. However, to maintain accuracy, documents claiming expenses at oriental medical clinics and pharmacies were excluded. Nonetheless, there is a probability of misclassification due to inaccurate data, and the number of examinees of 100,000 individuals might not be accurate and might not reflect the true occurrence rate of DH. However, the objective of this research was not to calculate the accurate occurrence rate of DH but to identify the differences or tendencies according to age, sex, and health insurance eligibility; therefore, this inaccuracy might not pose a problem. Despite these limitations, the study findings are relevant because we identified the differences in DH occurrence among the citizens of a country according to labor tasks. 

## 5. Conclusions

The number of individuals diagnosed with symptomatic CDH and LDH increased with age, and the incidence of these diseases was higher in women than in men. Additionally, the incidence differed depending on the subject’s qualification for health insurance. These findings may help identify the association between DH and tasks performed at the workplace. Further research on distinctions of DH occurrence according to a specific business category or occupation and varied task burdens is needed.

## Figures and Tables

**Table 1:** Mean number of subscribers according to gender, age and health insurance eligibility (2004–2010) (person).

Gender	Age	Region	Company	Government Office and Private School	Medical Care	*p*-Value *
Male	20–29	281,506	1,169,017	136,223	20,221	<0.001
	30–39	1,135,868	2,287,096	285,904	42,423	
	40–49	1,759,028	1,710,430	321,634	120,896	
	50–59	1,142,273	881,303	213,928	103,216	
	60–69	570,752	342,511	18,992	78,225	
Female	20–29	213,898	1,178,690	150,430	145,31	<0.001
	30–39	485,794	923,692	211,537	55,115	
	40–49	661,224	695,133	146,633	99,192	
	50–59	411,267	332,118	51,620	67,926	
	60–69	268,635	91,770	3988	101,613	

* Results of Chi-square test according to age, eligibility.

**Table 2:** Number of patients with CDH of 100,000 individuals according to gender, age, and health insurance eligibility (2004–2010).

Gender	Age	Region	Company	Government Office and Private School	Medical Care	*p*-Value
Male	20–29	187.87	229.29	205.34	186.51	
	30–39	351.90	466.49	449.85	465.38	
	40–49	659.89	882.93	828.85	889.43	
	50–59	1023.90	1288.37	1187.45	1153.89	
	60–69	1227.78	1438.70	1812.04	1137.38	
	Total	3451.34	4305.78	4483.53	3852.59	<0.001 *
Female	20–29	224.07	55.00	222.69	291.99	
	30–39	492.10	534.39	428.16	739.23	
	40–49	1040.88	1179.16	994.90	1527.05	
	50–59	1522.20	1838.00	1548.13	1871.57	
	60–69	1422.27	1679.67	2460.95	1405.89	
	Total	4701.52	5286.22	5654.83	5835.73	<0.001 *

* Results of the Chi-square test according to total number of eligibility.

**Table 3:** Number of patients with LDH of 100,000 individuals according to gender, age and health insurance eligibility (2004–2010).

Gender	Age	Region	Company	Government Office and Private School	Medical Care	*p*-Value
Male	20–29	877.42	1179.71	1059.08	965.76	
	30–39	1061.13	1325.50	1156.03	1433.18	
	40–49	1216.15	1465.05	1276.52	1959.42	
	50–59	1566.40	1815.45	1621.91	2321.48	
	60–69	2194.27	2374.11	2927.55	2374.65	
	Total	6915.37	8159.82	8041.09	9054.49	<0.001 *
Female	20–29	716.90	868.97	698.85	1023.43	
	30–39	1125.99	1151.90	924.05	1784.32	
	40–49	1780.81	1932.13	1296.16	2819.21	
	50–59	2640.41	3225.92	2037.97	3618.64	
	60–69	3472.58	3876.77	4323.69	3451.75	
	Total	9736.69	11055.69	9280.72	12697.35	<0.001 *

* Results of Chi-square test according to total number of eligibility.
